# Bedtime Smart Phone Usage and Its Effects on Work-Related Behaviour at Workplace

**DOI:** 10.3389/fpsyg.2021.698413

**Published:** 2021-08-18

**Authors:** Abida Ellahi, Yasir Javed, Samina Begum, Rabia Mushtaq, Mobashar Rehman, Hafiz Mudassir Rehman

**Affiliations:** ^1^Department of Management Sciences, Abbottabad University of Science and Technology, Abbottabad, Pakistan; ^2^Higher Colleges of Technology, Abu Dhabi, United Arab Emirates; ^3^School of Management Sciences, Quaid-i-Azam University, Islamabad, Pakistan; ^4^Faculty of Information and Communication Technology, Tunku Abdul Rahman University, Kampar, Malaysia; ^5^Faculty of Business and Finance, Tunku Abdul Rahman University, Kampar, Malaysia

**Keywords:** smart phone usage, sleep quality, work performance, interpersonal conflicts, work engagement, bedtime, sleep disorder

## Abstract

The over usage and over dependency on digital devices, like smartphones, has been considered as a growing international epidemic. The increased dependency on gadgets, especially smartphones for personal and official uses, has also brought many detrimental effects on individual users. Hence it is vital to understand the negative effects of smartphone usage on human. Therefore, this study aims to investigate the effects of bedtime smartphone usage on work performances, interpersonal conflicts, and work engagement, *via* the mediating role of sleep quality among employees. Using a cross-sectional study design, a questionnaire-based field survey was conducted on 315 employees who participated as respondents. The results confirmed the negative effects of bedtime smartphone usage on sleep quality. Along with it, the effects of sleep quality on work performances, work engagements and interpersonal conflicts were also proven to be statistically significant. Regarding the mediating role of sleep quality, it was empirically evident that sleep quality mediates the relationship between bedtime smartphone usage with work performances and interpersonal conflicts. The findings revealed that bedtime smartphone usage reduces sleep quality among the employees, resulting in lower work performances and engagements while contributing to higher interpersonal conflicts. The findings concluded that smartphone usage before sleep increases the prospects of employees to be less productive, less engaged, and have more workplace conflicts. The findings warrant the continued managerial as well as academic research attention, as the smartphones are now used by many organisations to run businesses as well.

## Introduction

The technological revolution has made smartphones to be exceptionally appealing and well-known. They have become an important piece of daily life, being progressively utilesed among the kids and adults. The smartphones are one of the most widely recognised methods for corresponding and communicating; therefore, they are becoming a necessity in the humans' lives. For most populations, a smartphone is a primary thing human will use when awake in the morning and before they sleep. However, the excessive usage of mobile phones has been associated with many health problems, including both biological and psychological issues. For example, Gugushvili et al. (2020) concluded that problematic or excessive smartphone usage is negatively associated with the emotional well-being of users. Moreover, it has been reported that obsessive usage “*leads to mental health symptoms such as sleep disturbance and depression*” (Thomée et al., 2011; Lee et al., 2014). Sleeping routine affects the performances (Dewald et al., 2010) in the human body, while sleep disorders can cause many physical and psychological problems.

Even though the smartphone usage may contribute to certain benefits, but the continuing usage of the smartphone at home and after the work may inhibit the employees “*to fully recover from work activities while away from the office, especially at night. Employees often use smartphones for work within an hour of going to bed, and many sleeps within reach of their smartphones*” (Lanaj et al., 2014). This causes insufficient sleep that weakens many cognitive functions, “*including memory, concentration, and attention*,” which are vital for everyday activities (Ellis et al., 2014). Due to these issues, the problems related to work engagements may arise at the workplace the very next day or in the near future (Binnewies et al., 2009).

The previous study by Rehman et al. (2019) observed that the smartphone usage at the workplace might decrease the work performances. Regardless of the subjective evidence showing the impacts of the smartphone on the employees' subsequent sleep and functions at work, unfortunately, these propositions were not empirically confirmed (Lanaj et al., 2014). Moreover, there has been a scarcity of research exploring the links between the bedtime usage of smartphones with sleep quality in adults, both locally and globally (Alshobaili and AlYousefi, 2019).

Overall, the literature suggests that the research related to smartphone usage is scattered, particularly in psychology, medical, education, and technology topics. The specific number of research related to smartphone usage in the management or organisational behaviour domain is very less, as most have focused on work-related use of smartphones and its consequences instead (Panwar and Agrawal, 2021). However, this study aimed to contribute to the existing information technology and organisational behaviour literature, while providing insights into smartphone usage and its linkage with the employees' workplace behaviour.

Keeping in mind the gap identified in the literature, this research aims to provide a view of employees' workplace behaviour due to bedtime smartphone usage and the results on sleep quality. In the current study, the authors sought to examine the associations between the bedtime smartphone usage with the workplace behaviour of employees, by taking into effects their sleep quality. The first objective of the current study is to examine the link between smartphone usage at bedtime the sleep quality among employees. In this regard, it was predicted that bedtime smartphone usage would be associated with poorer sleep quality among the employees. The second objective is to examine the associations of poor sleep quality with certain workplace behaviour like interpersonal conflicts, work engagements and performances at work among the employees. The third objective is to explore the mediating role of sleep quality among the relationships of bedtime smartphone usage with the work performances, work engagements, as well as the interpersonal conflicts among employees. The following research questions have been formulated to guide the study objectives:

RQ1: How does bedtime smartphone usage influence sleep quality of employees?

RQ2: What is the effect of sleep quality on employee work performance, work engagement and interpersonal conflicts at work place?

RQ3: How does sleep quality intervene between bedtime smartphone usage and employee work performance, work engagement and interpersonal conflicts at work place?

The study is unique in the sense that it aims to measure the effects of bedtime smartphone usage by the employees and on their work-related behaviour. Three useful behaviour indicators such as work performances, work engagements, and interpersonal conflicts have been selected for the work-related behaviour analysis. To the authors' knowledge, all these three consequences have not been previously explored all together and in relation to the bedtime smartphone usage and sleep quality among the employees. Moreover, in particular, the interpersonal conflicts have not been taken into account for the analysis of bedtime smartphone usage and sleep quality. Hence, the study makes important contributions to many research domains, such as information technology, psychology, organisational behaviour and mental health. The study directs the implications of increased smartphone use, especially at bedtime, which is silently bringing some negative consequences into the workplace. Thus, the study provides insights into the influences of smartphone usage and the implications for the organisation.

## Literature Review

### Underpinning Theory–Media System Dependency Theory

The media dependency theory in the field of communication sciences was first developed by Ball-Rokeach and DeFleur (1976). The theory is a systematic way to investigate the impacts of media on audiences and to determine the associations between media, audience, and social frameworks. The principle focal point of the media dependency theory is the connexion between the media and the audience. Similar to the current information and knowledge-based societies, people general build up a reliance on the media in order to fulfil their necessities. One of the media dependence theory propositions states that the more the functions are served by a media, the more the audience's dependency on the medium. As the dependence on media grows, the media gains more importance and consequently having stronger effects on the individuals (DeFleur and Ball-Rokeach, 1989). The dependency on media brings out three effects, i.e., cognitive, affective and behavioural effects. In the current study, the media dependency theory is used to support the theoretical framework of the study. By drawing from the media system dependency theory, this study investigates how bedtime smartphone usage (dependency) brings the cognitive outcome (work performances and work engagements), affective outcome (work engagements), and behavioural outcome (interpersonal conflicts).

### Bedtime Smartphone Usage and Sleep Quality

Living without a smartphone is unthinkable for any adolescents. On the one hand, smartphone facilitates the ways of life, but on the other hand, it also leads to mental and physical problems (Choi et al., 2012). The increasing frequency and time spent on the smartphone lead to smartphone addiction (Lee et al., 2014). Smartphone addiction is a non-chemical behavioural addiction that involves human-machine interactions, also known as technological addictions (Lin et al., 2014). Recent studies show that smartphone addiction is related to sleep disturbance and depression (Perlow, 2012). The addiction leads to sleep interference as the smartphones are placed within reach even when sleeping at night (Perlow, 2012).

The media system dependency theory also highlights this association between the media devices with its users. According to Park et al. (2013). “*The basic claim of the media-system dependency theory is that, on an individual level, as a person becomes more dependent upon media use in order to fulfil their goals, the media would have more influences on him or her. This definition and proposition of media dependency are well-suited to account for the use of smartphones with their enhanced functions and various features. That is, smartphones enable users to achieve many of the important goals in their everyday lives. If the functions and features make people find smartphones useful, easy to use, and eventually lead them to use, they are likely to become dependent on smartphones.”* Previously, smartphone usage is associated with decreased mental health emotional well-being (Elhai et al., 2019). However, the recent area showing that use of smartphone is the main reason for sleep problems in adolescence has gained much attention in the past few years (Rafique et al., 2020).

In previous literature, many studies found that smartphone overuse at nighttime has a virtual role in sleep quality (Rod, 2018). Most companies provide smartphone to their employees for instant access to work emails and documents, thus also providing connectivity to work (Carlson, 2012). The employee connectivity with work while away from the office and after the working hours affect the rest and recovery times from a hectic workday (Huhman, 2011). The studies showed that most of the individuals that are using their mobiles at the hour before going to bed are actually trading off their sleeping time. This trade-off between the smartphone usage with the sleeping time leads to lower quality of sleep, hence failing to be active on the following workday (Sonnentag et al., 2008). According to Brunborg et al. (2011), the smartphone is an active contributor to the poor quality of sleep. Therefore, from the literature, the first hypothesis is drawn as bedtime smartphone usage by employees leads to poor sleep quality. Thus, the first hypothesis drawn is:

**Hypothesis 1:***Bedtime smart phone usage will be negatively related to sleep quality among employees*.

### Work Performance

Rasool et al. (2019) pointed out that the employee productivity is caused by multiple factors like “*working environment, supervision, individual abilities, and an integrated motivational set of policies and organisational standard operating procedures.”* This leads to the clue of individual-level factors like sleeping habit, that can affect the employee's performances at work. The consequence of “*employee sleep quality*” includes the effects on the work performances for the following day. The human cognitive process is affected by low sleep quality, therefore having adverse effects on the problem-solving and complex decision making (Killgore et al., 2006).

There is sufficient evidence that individuals with better sleeping quality conduct fewer workplace errors due to better alertness and concentration (Killgore et al., 2008). Lawson and Lee (2018), in their study conducted among the nurses, found that good sleeping quality leads to high work productivity. Thus, sleep quality is important for being productive at work. In another study, Laethem et al. (2019) studied sleep quality as a mediator for some variables, including the self-reported work performances variable. Similarly, the study also noticed that the sleep quality and its relation with job demands and performances had not been investigated properly and thus, has been ignored.

In contrast, one stream of research supported smartphone usage at the workplace for enhancing work performances. For example, Pitichat (2013) argued that smartphone usage in the workplace ease organisational communication and collaboration, increasing work efficiency and productivity. However, another stream of research points out that the increasing use of smartphone may bring unwanted consequences, such as smartphone addiction symptoms (Li and Lin, 2018). This can be harmful to the employees, as well as organisations because the concentration and productivity at work are reduced (Ray, 2015; Seals, 2015; Li and Lin, 2018). In support, Gugushvili et al. (2020) highlighted in their studies that the excessive smartphone usage is associated with negative outcomes, such as decreased productivity (Duke and Montag, 2017), distraction from the learning process (Rozgonjuk et al., 2018), and low-quality communication in the social settings (Vanden Abeele et al., 2016).

In the light of media system dependency theory, the cognitive outcomes result in the increasing media use. Therefore, it can be argued that the employees are becoming dependent on their smartphones, either for work or personal motives and use it even at bedtime. This regular usage of smartphone at bedtime ultimately reduces their sleep quality, bringing cognitive effects in terms of reduced work performances. Pilcher and Morris (2020) confirmed that poor sleeping quality negatively impacts the motivation to perform well at the workplace, while the sleep-deprived employees show decreased work performances and increased work-related errors. Low sleep quality alters the cognitive arousal, leading to the absence of mind, subsequently increases the chances of workplace errors. Therefore, it is predicted that sleep quality as a result of smartphone usage is positively related to employees' workplace performances.

**Hypothesis 2:***Employee's sleep quality will be positively related to workplace performance among employees*.

**Hypothesis 2(a):***Employee's sleep quality will mediate the effect of bedtime smart phone usage on workplace performance among employees*.

### Interpersonal Conflict

There has been growing attention in the organisational setting to study the job-related psychological stressors. One of the serious job stressors is the interpersonal conflicts in the workplace. The interpersonal conflicts are defined as the negative clashes with others at the workplace, for examples by taking part in contentions with an associate or being treated in a terrible way by the superior (Spector and Jex, 1998). According to Zhou et al. (2020), human interactions at workplaces are commonly bringing both the positive and negatives dimensions, leading to several types of outcomes.

The interpersonal conflicts are resulted from the human interactions and have been proven to the causes of many health outcomes like depression, burnout and insomnia. However, the effects of these conflicts still “*remains neglected in sleep literature*” (Sakurai et al., 2014). Many studies highlighted the interpersonal conflicts as the factors affecting sleep quality. For example, Chen and Li (2019) found that the workplace ostracism arises from stressful interpersonal relationships are negatively affecting sleep quality. Most of the research stream focused on sleep quality or insomnia as an outcome of many work-related factors (Nauman et al., 2019). However, the literature does not expand on the events that happen after insomnia and poor sleep quality, especially in the context of interpersonal conflicts. In one of the few studies, Jha and Jha (2010) studied four antecedences of interpersonal conflicts, i.e., individual differences, interpersonal issues, organisational factors, and extra organisational issues, to determine the potential sources of interpersonal conflicts at the workplace. The extra organisational factors that can trigger interpersonal conflicts need to be verified through empirical surveys, as pointed out by Jha and Jha (2010).

Overall, it is difficult to find the literature that studies the associations between sleep quality as a result of smartphone usage with its role in the interpersonal conflicts among employees at the workplace. Therefore, this study is filling this gap by observing that individuals with lower quality sleep encounter affective and cognitive impairments that lead to conflicting behaviours. The media system dependency theory also highlights the behavioural outcomes of increased media usage. In this study, the behavioural outcome as a result of bedtime smartphone usage is set as the interpersonal conflicts shown by the employees at their workplace as a result of poor sleeping quality. Accordingly, the interpersonal conflicts increase when the workplace violence and occupational stresses are increasing, resulting in poorer work performances (Rasool et al., 2020).

Simon et al. (2020) argued that poor sleep quality impacts the employees' societal functioning that even moves beyond the interpersonal or workplace relationships. There is cumulative evidence that showed that the low sleep quality is adversely affecting the mood and psychopathology symptoms, and the sufferers are more likely to respond to frustrating circumstances and other affective processes (Scott et al., 2006). Hence, the hypothesis drawn is that the employees' sleep quality is negatively related to interpersonal conflicts among employees and mediate the effects of late-night smartphone use on interpersonal conflicts.

**Hypothesis 3:***Employee's sleep quality will be negatively related to interpersonal conflicts among employees*.

**Hypothesis 3(a):***Employee's sleep quality will mediate the effect of bedtime smart phone usage on interpersonal conflicts among employees*.

### Work Engagement

The personal energies of individual invested in work roles represent their work engagements (Rich et al., 2010). The work engagement of an employee is considered vital for employees and organisations, as engaged employees feel more dedicated, energetic, motivated and productive, which ultimately leads to the better organisational performances (Van Laethem et al., 2018).

The researchers argued that the activities performed the previous day affect the engagement level on the next day. So, the employees who use their smartphone at bedtime and have poorer sleep quality are deemed to have lower work engagements (Lanaj et al., 2014). The sleep quality has implications for the work engagements because the lower sleep quality affects the focus and efforts entailed in engagements (Bakker and Leiter, 2010). Derks and Bakker (2014) poignantly argued that “*by introducing the smartphone into our private domains, the stressor of work has become salient in our living rooms. It is plausible that the smartphone with its request for 24/7 availability disturbs the important process of disengaging from work and recovery.”* Van Laethem et al. (2018) in their study examined and assessed the employees' work-related smartphone use, psychological detachment after work, and engagement during work. Their study indicated some negative connexions between employee smartphone usage with work engagements. Lin et al. (2020) in their study found increase in employee engagement due to increase in smartphone usage.

The media system dependency theory also supports this, as when the individuals are using a medium more frequently, they are also experiencing greater cognitive, behavioural and affective outcomes, because the work engagement is an affective state (Schaufeli et al., 2002). Therefore, the work engagements as an affective outcome of smartphone usage at bedtime are appropriate to link to the media dependency theory. Park et al. (2013) also supported that the definition and proposition of media system dependency theory are the best fit for the usage of the smartphone. Therefore, it is expected that the high (or low) quality sleep leads to high (or low) work engagements because the energy needed for engagement is vulnerable to the sleeping quality (Baumeister et al., 2007) that is affected by bedtime smartphone usage. Consistent with the arguments presented above, the following hypothesis was formulated.

**Hypothesis 4:***Employee's sleep quality will be positively related to work engagement*.

**Hypothesis 4(a):***Employee's sleep quality will mediate the effect of bedtime smart phone usage on work engagement*.

**Figure 1:**
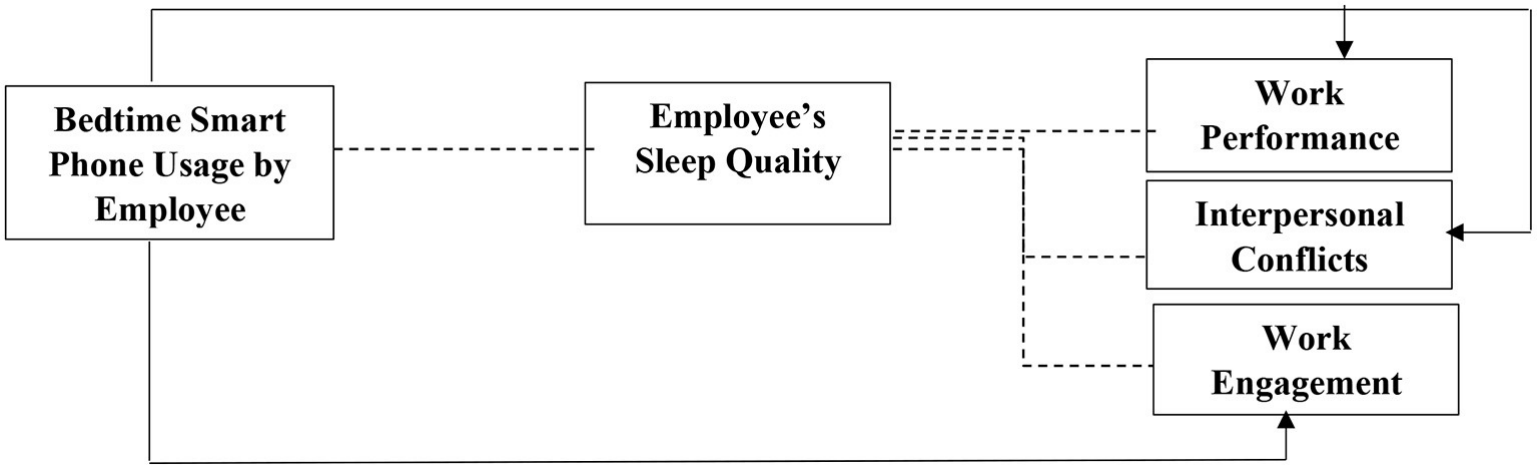
Research model. Solid lines represent direct relationships whereas dashed lines represent indirect relationships.

Please refer to Figure 1 for complete research model.

## Research Methodology

### Research Approach

An online survey was conducted among the employees working in both the private and public sectors in Pakistan to test the hypotheses used in this study statistically. The online survey method was used for several reasons. First, it is a low-cost but easy method to collect the necessary data. Second, the response rate is usually higher than the manual distribution of the questionnaires. The online survey offers the advantage of being cost effective, easily accessible and time saving (Sudha et al., 2018). Third, most of the data collection was done during the COVID-19 pandemic period, where the majority of the employees were observing the lockdown, or they were working from home. Hence, the online survey was the most appropriate strategy to collect the responses. The respondents could not be accessed in their physical office buildings. However, most of the respondents filled the questionnaire during office hours. The questionnaire took ~15min to complete. The study was a cross-sectional study in nature and was conducted from January 2020 to May 2020 in Pakistan, based on a convenience sample approach. The convenience sampling technique is easy to use, and aids in data collection without complexities when using random sampling techniques (Samma et al., 2020).

### Sample and Procedure

The study was approved by the ethical committee of the authors' University, for which a prior approval was obtained by authors. The questionnaire contained the introduction and purpose of survey, as well as the statement of confidentiality of responses. There was no separate consent statement in the questionnaire and the successful as well as complete return of questionnaire was considered the respondents' consent. The sample size was calculated using the sample size formula mentioned in the study of Srivastav et al. (2021). The sample size (n) was calculated according to the formula: *n* = z^2^^*^ p ^*^ (1 – p)/d^2^, where *z* = 1.96, *P* = 90% as the response rate of the online survey, and *d* = 0.05. This calculation gave the minimum sample size of *n* = 139. According to Sekaran and Bougie (2016), when population size ranges from 10 to 1000000, the sample size should be from 10 to 384 (Table 11.3, p.294). By keeping this mind, an initial sample size of 350 respondents was used in this study. Out of these 350, only 315 useful and complete responses were used for analysis purpose. The 35 responses were ignored due to missing values and with same response for all items, which can affect the quality of research. This made a 90% response rate of this online survey. The employees from both the public and private sectors were targeted.

The selected respondents were from both the service and manufacturing sectors of Pakistan. In the service sector context, the respondents were from the educational institutes, banks, and visa processing companies. Meanwhile, the respondents from the manufacturing sector were working in food and beverages companies. This sampling choice reflected the diversity of employees' population, as these selected respondents were from different service and manufacturing sectors, hence, it was a help in better understanding of the employees' behaviour due to late-night smartphone usage. Additionally, this sampling population supported the advocate by Gurven (2018) that poignantly stated the use of sample diversity as “*nontraditional samples—that is, moving beyond students, cities, and western borders—are critical for generating a broader understanding of human nature and its multifaceted psychological and behavioural manifestation*.” The targeted sample was approached via the social media networks, e.g., Linkedin, Whatsapp and Facebook. Similarly, email was also used to contact respondents. The URL link of google form was shared with the targeted sample. As the data collection was conducted online, hence, there was no restriction in term of office timings. Few of the respondents filled the questionnaire on the same day of contact, however, majority of them took maximum of one day time and returned the questionnaire the next day. As majority of them were working online, therefore, they did not take much time to fill the online questionnaire. However, the reminder link was also sent to them who could not respond on the day, they committed to respond.

### Instrument Development

A 30-questions survey questionnaire was used as an instrument for data collection. The questionnaire was adopted from previous studies. The questionnaire was designed using Google form; then the link was shared among the participants to get quick and timely responses. The 30 questions related to the independent and dependent variables were asked. In addition, the questionnaire also included the respondents' background information like age, gender, the years of experience, smartphone usage at bedtime, and the purpose of using smartphone at bedtime.

At first, a pilot study consisted of 15 participants was conducted to ensure the reliability and validity of the instrument. The sample size of 15 participants for pilot study is supported by the Julious (2005) who recommended a minimum of 12 sample size for pilot study due to “feasibility; precision about the mean and variance; and regulatory considerations.” The questionnaire was also reviewed by six subject experts who swotted the format and wordings to ensure the eligibility of the questionnaire in measuring the intended constructs. These experts were having doctorate degree and were involved in conducting various research projects. Out of these six experts, two were females and four were males. Hence, the face validity of the instrument was also confirmed. The I-CVI values are mentioned in Appendix. After that, a few changes were made based on the suggestions by the subject experts. The questionnaire filled in this pilot study was analysed using SPSS software to ensure the reliability. Cronbach alpha was used as a reliability coefficient to assesses the consistency of the selected scale. The accepted lower limit for Cronbach alpha is 0.70, however, 0.60 is also acceptable in exploratory studies (Hair et al., 2007). The Cronbach alpha values of the variables Smart Phone Usage (α = 0.71), Sleep Quality (α = 0.71), Work Performance (α = 0.74), Interpersonal Conflicts (α = 0.70), Work Engagement (α = 0.78) met the required limit.

### Variable Measures

The independent variable, i.e., bedtime smartphone usage, was measured by using four items that were adopted based on the studies reported by Alshobaili and AlYousefi (2019) and Lanaj et al. (2014). The purpose was to measure the smartphone usage of the respondents at bedtime and before they are sleeping. The items were measured on a 5-point scale (where 1 = Almost always to 5 = Never). The items included (1) “How frequently do you use your smartphone at bedtime (before sleeping)” and was measured as (1 = 15 min or less, 5 = more than 60 min), (2) “How many time do you spend using a smartphone at bedtime (before sleeping) and was measured as (1 = strongly agree, 5- strongly disagree), (3) “I cannot resist using Smartphone at bedtime (before sleeping)” and was measured as (1 = 1–3 times per month, 5= Every day) and (5) “How many times you use your smartphone at bedtime (before sleeping) in a month.”

The mediator variable, i.e., sleep quality, was perceived by the smartphone users and was assessed using the Sleep Athens insomnia scale taken from the study of Monterrosa-Castro et al. (2016). The responses were measured using a 5-point scale (e.g., 1 = No problem to 5 = did not sleep at all). The scale was used to measure eight items like “Sleep induction (the time it takes you to fall asleep after turning-off the lights),” “Awakenings during the night,” “Final awakening earlier than desired,” “Total sleep duration,” “Overall quality of sleep (no matter how long you slept),” “Sense of well-being during the day,” “Functioning (physical and mental) during the day,” and “Sleepiness during the day.”

The first dependent variable, i.e., work performance, was measured using five items used in the study of Hanaysha (2016). The scale of 1 = Strongly Agree to 5 = strongly disagree was used to measure the items such as “I accomplish tasks quickly and efficiently,” “I accomplish tasks quickly and efficiently,” “I have a high standard of task accomplishment,” “My work outcomes are of high quality,” and “I always beat our team targets.”

The second dependent variable, i.e., interpersonal conflict at work, was analysed using four items. Similarly, the responses were measured using a 5-point scale (1 = less than once per month or never to 5 = several times per day). The four items were adopted from the study of Eatough (2010), and consisted of “How often do you get into arguments with your co-workers at work,” “How often does your co-workers yell at you at work,” “How often is your co-workers rude to you at work,” “How often does your co-workers do nasty (bad) things to you at work.”

The third dependent variable, i.e., work engagement, was measured using the items adopted from the study of Hanaysha (2016). The responses were recorded on the scale of 1 = strongly agree to 5 = strongly disagree. This variable had 14 items such as “When I am working, I forget everything else around me” and others.

## Results

### Demographic Characteristics

Table 1 contains the respondents' demographic information. The values showed that the majority of the individuals are between the ages of 18–25 years, while the second-highest number of individuals are between the ages of 26–35 years. Only a small percentage of individuals are above the age of 46. Surprisingly, none of the respondent fell in the age group of 36–45 years. The number of respondents was evenly split between males and females. Most of the individuals are working in the public service sector. Then, 46% of the respondents have a working experience of 1–2 years. Moreover, 90% of the individuals responded that they use a smartphone for connectivity purpose. Meanwhile, only a small portion of the respondents use smartphones at bedtime to visit social networking sites, while the majority use it for entertainment and web surfing. More than half of the respondents felt that they are addicted to their smartphones.

**Table 1:** Survey participants' characteristics.

**Characteristics**	**Category**	**Number**	**Percentage**
Age	18–25 years	171	54.3
	26–35 years	131	41.6
	36–45 years	0	0
	46 years and above	13	4.1
Gender	Male	173	54.9
	Female	142	45.1
Which sector do your work for?	Public	266	84.4
	Private	49	15.6
Sector of employment	Service	266	84.4
	Manufacturing	49	15.6
Work experience	1–2 years	146	46.3
	Between 2 and 5 years	72	22.9
	More than 5 years	97	30.8
Do you use smart phone for connectivity	Yes	283	89.8
	No	32	10.2
Do you use Smart phone at bedtime	Yes	294	93.3
	No	21	6.7
Purpose of smart phone use at bedtime	Social networking sites	9	2.9
	Entertainment	112	35.6
	Web surfing	148	47.0
	Other	46	14.6
Do you feel yourself smartphone addicted?	Yes	171	54.3
	No	110	34.9
	Don't know	34	10.8

### Descriptive Statistics

At first, the data normality was checked in SPSS software. In order to check the normality test, there were two tests, Kolmogorov–Smirnov and Shapiro–Wilk. According to Mishra et al. (2019) “the Shapiro–Wilk test is more appropriate method for small sample sizes (<50 samples) although it can also be handling on larger sample size while Kolmogorov–Smirnov test is used for n ≥50.” The calculated values in SPPS showed that the significant values were more than 0.05, confirming the normal distribution.

The descriptive analysis in Table 2 indicates the Pearson correlation, mean (*M*) and standard deviation (*S.D*). The Pearson correlation checks the linear relationship between variables derived from a normal distribution with no significant outliers. Hence, it is a frequently used parametric test. The normally distributed measurements can be better treated with parametric test. All values confirmed a positive correlation among variables. The lowest correlation was between low sleep quality and interpersonal conflict (*r* = *0.5*2^**^), while the highest correlation was found between smart phone usage and interpersonal conflict (*r* = *0.87*). The table also reported mean and standard deviation values. The values show that for variable smartphone usage (*M* = 2.80, *S.D* = 0.59), sleep quality (*M* = 2.07, *S.D* = 0.71), work performance (*M* = 2.37, *S.D* = 0.72), interpersonal conflict (*M* = 2.08, *S.D* = 0.95), and for work engagement (*M* = 2.34, *S.D* = 0.64).

**Table 2:** Pearson correlation, mean, standard deviation.

	**Variables**	**1**	**2**	**3**	**4**	**5**	**M**	**S.D**
1	Smart phone usage	1	0.64	0.65	0.87	0.76	2.80	0.59
2	Sleep quality		1	0.63	0.52	0.69	2.07	0.71
3	Work performance			1	0.82	0.64	2.37	0.72
4	Interpersonal conflicts				1	0.70	2.08	0.95
5	Work engagement					1	2.34	0.64

### Construct Reliability and Validity

The analysis was performed using the Smart PLS software, robust software to evaluate the reliability and convergent validity of the model. Table 3 shows the values from the Cronbach's alpha, composite reliability (CR) and the average variance extracted (AVE) analyses. These values were calculated using PLS Algorithm in smart PLS software. The values of AVE should be higher than 0.5 (Becker et al., 2018). Meanwhile, the values of Cronbach's alpha and composite reliability (as shown in Table 3) were shown to be above 0.06 and revealed high reliability of the items selected. Hence, the reliability of the scale items was also confirmed. In order to check the discriminant validity, the Heterotrait-Monotrait ratio of correlations (HTMT) was also checked, as shown in Table 3. The Heterotrait-Monotrait is a new technique of assessing discriminant validity and is found having superior performance than other techniques (Henseler et al., 2015). The values showed that the HTMT ratio values of the constructs were below the threshold value of 0.85. Hence, the discriminant validity of items was confirmed in this study.

**Table 3:** Construct reliability and validity.

	**HTMT Ratio**								
	**Construct**	**Alpha**	**CR**	**AVE**	**1**	**2**	**3**	**4**	**5**
1	Bedtime SP usage	0.631	0.653	0.531					
2	Sleep quality	0.784	0.844	0.620	0.261				
3	Work performance	0.782	0.849	0.532	0.393	0.453			
4	Interpersonal conflict	0.755	0.841	0.676	0.295	0.399	0.513		
5	Work engagement	0.872	0.892	0.757	0.215	0.304	0.537	0.782	

### Hypotheses Testing

After the descriptive, correlation, reliability and validity analyses were carried out, the hypotheses were tested using the Smart PLS 3.3.2 software. The hypotheses were tested using the bootstrapping process based on 1,000 subsamples, as recommended by Hair et al. (2017). The Table 4 contains values of unstandardized estimates (β), critical ratio (also called t-statistic), *p*-values, and 95% confidence interval bias corrected (CIBC).

**Table 4:** Total, direct, and indirect effects.

	**Paths**	**β**	**t Statistics**	***P***	**95% CIBC**
**Total effects**					
1	Bedtime SU -> Sleep quality	−0.35	2.40	0.05	[0.377–0.47]
2	Bedtime SU -> Work performance	0.16	2.26	0.02	[0.106–0.119]
3	Bedtime SU -> Conflict	0.25	2.20	0.02	[0.204–0.244]
4	Bedtime SU -> Engagement	0.24	7.45	0.00	[0.153–0.207]
5	Sleep quality_ -> Work performance	0.43	8.72	0.00	[0.345–0.541]
6	Sleep quality_ -> Conflict	−0.32	5.69	0.00	[0.218–0.440]
7	Sleep quality_ -> Engagement	0.44	9.30	0.00	[0.357–0.543]
**Direct effects**					
1	Bedtime SU -> Work performance	−0.14	−0.18	0.75	[−0.187–0.258]
2	Bedtime SU->Interpersonal conflict	−0.06	0.50	0.52	[−0.124–0.213]
3	Bedtime SU -> Work engagement	0.03	−0.28	0.69	[−0.390–0.455]
**Specific indirect effects**					
1	Bedtime SU -> Sleep quality -> Work performance	0.15	2.204	0.02	[0.290–0.485)]
2	Bedtime SU -> Sleep quality -> Conflict	0.13	2.146	0.03	[0.415–0.553]
3	Bedtime SU -> Sleep quality_ -> Engagement	0.16	2.263	0.70	[−0.359–0.538]

The values of total, direct and indirect effects are as presented in Table 4. The total effects are without a mediator, the direct effects are with the mediator, and the indirect is through mediator variables. The mediation analysis was performed to assess the mediating role of sleep quality between the bedtime smartphone usage with the work performance, interpersonal conflicts and work engagement variables. The results in Table 4 revealed that the total effects of bedtime smartphone usage on work performances (β = 0.16, *p* = < 0.05), interpersonal conflicts (β = 0.25, *p* = < 0.05) and work engagements (β = 0.24, *p* = < 0.05) were significant. Similarly, the bedtime smartphone usage variable showed a significant but negative effect on sleep quality (β = −0.35, *p* = < 0.05). Thus, hypothesis 1 was supported. The impacts of sleep quality on work performances (β = 0.43, *p* = < 0.05), interpersonal conflicts (β = −0.32, *p* = < 0.05) and work engagements were also significant (β = 0.44, *p* = < 0.05). Therefore, hypothesis 2, 3, and 4 were also proven.

The direct effect showed that with the inclusion of sleep quality as the mediating variable, the impacts of bedtime smartphone usage on work performances (β = −0.14, *p* = >0.05), interpersonal conflicts (β = 0.06, *p* = >0.05) and work engagements (β = −0.03, *p* = >0.05) were insignificant. However, the indirect effect of bedtime smartphone usage on work performances (β = 0.15, *p* = < 0.05), interpersonal conflicts (β = 0.13, *p* = < 0.05) were found to be significant. Thus, these findings confirmed the mediation variable and supported the hypotheses 2(a) and 3(a). On the contrary, the mediating effect of bedtime smartphone usage on work engagements was not significant (β = 0.16, *p* = >0.05), as the upper and lower confidence interval values fall between zero. Therefore, the hypothesis 1, 2, 2(a), 3, 3(a), and 4 were accepted.

The effect size of the model is shown in Table 5. According to the guideline of Cohen (1988) small (0.02), medium (0.15), and large (0.35) ranges were used to measured the effect sizes of variables. More results are attached as Appendix.

**Table 5:** Effect sizes.

		**1**	**2**	**3**	**4**	**5**
1	Bedtime SP usage			0.240		
2	Interpersonal conflict					
3	Sleep quality		0.161		0.296	0.243
4	Work engagement					
5	Work performance					

## Discussion

The organisational success is reliant on an individual's performance. However, there are some elements that can disrupt the employee's output for an organisation. The aim of this research is to determine the effects on workplace behaviour by examining the within-person linkages between the bedtime smartphone usages with the sleep quality of employees.

The media system support theory was used to provide theoretical supports to the hypothesised relationships among the variables. On the basis of this theoretical support, the authors examined the impacts of direct and indirect effects on the individuals work outcomes, like work performances, interpersonal conflicts and work engagements. It has been viewed that the smartphone use at night results in lower working capacity, especially on the next day working capacity. For these reasons, it is essential to examine the influences of smartphone usage at bedtime on work-related behaviour.

The gap in the literature that has been declared by Alshobaili and AlYousefi (2019) was filled in this research, as the influences of smartphone usage at night on sleep quality were empirically examined. The results revealed that bedtime smartphone usage is a risk to the sleep quality of employees. These results were concurrent with the existing literature. The prior study concluded that smartphone use at bedtime reduces the students' sleep quality in Saudi Arabia, resulting in lower energy levels during the next day (Alosaimi et al., 2016). Similar research was conducted on the medical students of India and demonstrated that poor sleep quality is due to bedtime phone usage (Yogesh et al., 2014). The late-night smartphone use for work and the quality of sleep are specified as the signs that can show the reducing functions at work (Harris et al., 2020). The smartphone usage at bedtime consumes sleeping time and activates the mind processing through watching and reading contents on the phone.

The changes in sleep quality ultimately impact the worker performances, increase interpersonal conflicts and drops work engagements. The employees are having less sleeping quality because the smartphone usage at bedtime can reduce their work performances. Therefore, the employees may feel dizzy and tired during daytime and therefore cannot apply their working capabilities at full. This lowers the efficiency at work. Lesser quality sleep also increases the incidences of mood swings and can cause interpersonal conflicts between the employees. According to Swanson et al. (2011), the individuals who had reported a bad work outcome are having less sleep than others. The less sleep is linked with the deficiency at work performance and the difficulty to concentrate properly. Such employees also avoid social interaction with others, are more aggressive and cause conflicts easily. Gordon and Chen (2013) also found that poor sleep causes unnecessary conflicts and deleterious outcomes. Hence, the current research findings demonstrated the importance of quality sleep and supporting the needs to control smartphone usage at bedtime for better work performances and reduce the risk of conflict aversion.

Another negative effect of low sleep quality is low engagement at work by employees. Workers cannot focus and perform their work properly; hence, they did their work with less interest because of the lack of sleep from last night. The full mediation of work performances and interpersonal conflicts dependent variables supported that smartphone usage at bedtime can affect sleep quality, eventually affecting work performances and interpersonal conflicts. In conclusion, the effects of sleep quality on work engagements were proven. However, the mediating role of sleep quality between the relationship of bedtime smartphone usage and work engagements was not proven.

The study by Lanaj et al. (2014) highlighted the effects of “blue light” released by smartphones as “*this interferes with the production of melatonin,” … “which is a chemical that facilitates falling and staying asleep… using smartphones for work at night also interferes with employees' ability to detach and recover from work. Ruminating about impending deadlines or work responsibilities consumes cognitive resources leaving employees more depleted in the morning and less engaged at work the following day.”*

Therefore, in order to improve work performances and employee engagements, the employees must take proper sleep and do not waste time using smartphones late at night. Training should be provided to the employees with real-time examples to show them the importance of sleep for their health and office environment. Bedtime smartphone usage reduces the sleep quality of employees, while their work-related behaviours are also affected. This revelation is alarming for the employees, as well as the organisations. This is because the employees' performances are related to organisational performances and productivity. In the employees' context, their career growth and professional development could be affected if their work performances and work engagements are reduced, while the interpersonal conflicts are increased. These effects can be attributed to poor sleep quality as a result of bedtime smartphone usage. Therefore, this study highlighted the importance of investigating sleep quality as a result of technology gadgets usage. As the bedtime mobile phone usage was verified to impact sleep quality, future research should identify and examine deeper linkages among all these variables.

## Theoretical and Practical Implications

The study provides many implications in response to the media system dependency theory. For example, the study determined that two antecedents and individual-level factors, such as bedtime smartphone usage and sleep quality, were affecting the work-related behaviour of employees. These findings enhanced the comprehension of underlying processes, i.e., the smartphone usage at bedtime and sleep quality, can affect work-related behaviours like work performances, interpersonal conflicts, and work engagements. Additionally, the results extend the applications of media system dependency theory in the health-based employees' behaviour in the working settings, especially in technology usage during bedtime setting.

There are two streams of studies on smartphone usage. One stream is in favour of the usage of smartphone at work or for work. The usage brings positive outcomes in terms of enhancing work productivity, efficiency and enhancing work relationships, as it facilitates communication and information sharing between the employees (Demerouti et al., 2014). However, by favouring the positive outcomes of smartphones for work, it has led the organisations and employees to increase their dependency on them. The other stream of research is to brings forward the other side of smartphone usage, i.e., the negative outcomes. Although this stream has focused on many outcomes like medical, psychological or health-related effects, the work-related behaviour, especially in the context of employees, still requires more in-depth investigations, as many studies are focusing on the positive outcomes of smartphone usage for work instead. The research on the harmful effects of smartphone usage is still lacking or still few in number, especially the employees or organisational settings.

The role of media system theory states that the increasing dependence on any types of media, particularly, in this case, the dependency of smartphone posits many cognitive, affective and behavioural outcomes. The increased usage can lead to addiction, as well. In the background information of the questionnaire used in this study, the majority (54.3%, as shown in Table 1) of respondents also indicated that they are feeling addicted to their smartphone. The current research findings contribute to the media system dependency theory in many ways. First, the results of this current study provided empirical evidence to the applications of media system dependency theory in the organisational and employees work settings.

Second, the theory is further supported by highlighting a few of the cognitive, affective and behavioural outcomes. i.e., sleep behaviour, work performances, interpersonal conflicts and work engagements. Some studies had reported the link between the media system dependency theory with the work performances (Li and Lin, 2019), but the interpersonal conflicts are not being investigated with the media system dependency theory. Additionally, it has not been studied as an outcome of bedtime smartphone usage. Therefore, this study made significant contributions by exploring the interpersonal conflicts variable. Moreover, the previous researches mostly focused on the interpersonal conflicts as a factor of many organisational behavioural. This study has diverted research and theory attention by showing that interpersonal conflicts could be used as outcomes linked with bedtime smartphone usage and sleep quality. Thus, these findings could be new contributions to the affective, behavioural or cognitive outcomes of media system dependency theory.

Thirdly, the majority of the previous studies mostly focused on topics such as work-related smartphone usage at work and the work-related outcomes. Meanwhile, the researches related to smartphone usage for work at home or outside of work settings, and the effects on work-related behaviour are very limited (Lanaj et al., 2014). Moreover, the researches related to smartphone usage, regardless of work-related use or personal use, and the effects on work-related behaviour are even rarer. A smartphone is a personal gadget used not only for work but also for personal use. The background information analysis showed that the majority of respondents indicated that they use a smartphone at bedtime for web surfing (47%), for entertainment (35.6%), for visiting social networking sites (2.9%) and for other use (14.6) that was unspecified. The findings demonstrated that regardless of the purpose of using the smartphone at bedtime, it affects sleep quality and ultimately affects the work-related behaviour.

Fourth, as the media system dependency theory indicates only the cognitive, affective and behavioural outcomes, the study has expanded this notion by empirically confirming other outcomes like work performances, interpersonal conflicts and work engagements. The study has made a contribution to the theory by studying an indirect effect. As pointed out by Li and Lin (2019), future research needs to study the mediation effects in light of the media dependency theory and smartphone usage among the workers. Therefore, this study contributes to the media system dependency theory and fulfils the literature gap. It is important because the media system dependency can be used to study the direct and indirect effects of a certain media dependency. Along with it, the work performances, interpersonal conflicts and work engagements outcomes are able to bring more outcomes like turnover, absenteeism, satisfaction, or any other psychological and organisational variables. This indicates the potential room for developing more complex and more comprehensive models to study the media system dependency theory. Hence, this study has not only to extend the theory into the organisational settings, but it also highlights the potential applicability of this theory for further investigations.

### Managerial Implications

The study is useful for the human resources of companies, organisational behaviour studies, information technology, mobile health (m-health), as well as the psychological health domains. This research is important for the employees to realise the importance of sleep and to improve work performance or disturbed behaviour, by having a proper sleep at night and managing smartphone usage accordingly. Likewise, employers should also give special training programs on the importance of sleep to the employees to improve their personal and professional lives. For human resource management, the study posits a need to include the employees' personal habits or outside of work habits, like bedtime smartphone usage and sleep behaviour habits, in the training materials of employees. In addition, there has been an increasing dependency on smartphones for work, and the companies are now encouraging its use as well. Therefore, managers should also consider its positive and negative consequences. The findings of this study can also be used to alarm the organisations that if employees want to be productive at work, it is important for them to be aware of their personal life, especially in reducing the dependency on smartphones at bedtime and improving their sleep.

### Limitations and Future Directions

There are a few limitations of this study. Firstly, it has cross-sectional nature, as it does not provide a long-term change in the employees' behaviour due to the use of smartphone at bedtime. Secondly, the study is based on self-reported measures. Hence it can bring certain biases in the data. Thirdly, the research model did not account for any moderator variable, as sleep could be affected by other factors like age, gender or any other psychological states. Regardless of these shortcomings, the study gives a newer view of the impacts of cell phone use on sleep quality and other work-related behaviour. For example, Exelmans and Van den Bulck (2016) indicated that the moderator variables had been frequently disregarded in sleep and media studies.

The study is preliminary in nature, especially in the context of Pakistan. Therefore, this limitation is leaving enough room for future studies to extend the research model into various settings and to test with different techniques. For future studies, a longitudinal study is recommended in order to gain deeper insights into this phenomenon. Moreover, the future research should account for several other factors, such as gender and work type, that are not explored in this study to explore further linkages, so that more implications for the human resource and organisations can be confirmed.

## Conclusions

This research aims to provide workplace views of employees' behaviour due to smartphone usage during bedtime and to determine the consequences of employees' sleep quality. The verdict of this study findings revealed that the amount of smartphone usage should be given attention by employees, even outside of the office, especially during bedtime, as the amount was linked with lower sleep quality, which was associated with work-related ill behaviour. The employees should be extra vigilant, as this behaviour might unknowingly affect their career growth and progress since it is proven to be an inhibitor for work performances and engagement, as well as being the facilitator of their interpersonal conflicts at the workplace.

## Data Availability Statement

The raw data supporting the conclusions of this article will be made available by the authors, without undue reservation.

## Ethics Statement

The studies involving human participants were reviewed and approved by Abbottabad University of Science and Technology. The patients/participants provided their written informed consent to participate in this study.

## Author Contributions

AE was the first author who contributed to the idea and introduction section. YJ contributed to the overall proofreading of the manuscript and helped in incorporating all the changes suggested by reviewers. SB contributed to the literature review section of the paper. RM provided help during the data collection and data analysis phases. MR provided support to analyze and interpret the results. HR was responsible for proofreading and contributing to the discussion part of the paper. All authors contributed to the article and approved the submitted version.

## Conflict of Interest

The authors declare that the research was conducted in the absence of any commercial or financial relationships that could be construed as a potential conflict of interest.

## Publisher's Note

All claims expressed in this article are solely those of the authors and do not necessarily represent those of their affiliated organizations, or those of the publisher, the editors and the reviewers. Any product that may be evaluated in this article, or claim that may be made by its manufacturer, is not guaranteed or endorsed by the publisher.

## APPENDIX

**Table 6:** Questionnaire validity.

**Item description**	**Rater 1**	**Rater 2**	**Rater 3**	**Rater 4**	**Rater 5**	**Rater 6**	**Number agreement**	**I-CVI**	**S-CVI/Ave**	**Total agreement**
Item 1	4	4	4	4	4	4	6	1	0.96	28
Item 2	4	3	4	3	4	4	6	1		
Item 3	4	3	4	3	4	4	6	1		
Item 4	4	4	4	4	4	4	6	1		
Item 5	4	3	4	3	4	4	6	1		
Item 6	4	3	4	3	4	4	6	1		
Item 7	4	3	4	3	4	4	6	1		
Item 8	4	4	4	4	4	4	6	1		
Item 9	4	3	4	3	4	4	6	1		
Item 10	3	4	4	4	4	3	6	1		
Item 11	3	4	4	4	4	3	6	1		
Item 12	3	3	4	3	4	1	5	0.83		
Item 13	4	4	2	4	3	4	5	0.83		
Item 14	3	4	4	4	4	2	5	0.83		
Item 15	4	3	4	3	4	4	6	1		
Item 16	4	4	4	4	4	4	6	1		
Item 17	4	3	4	3	4	4	6	1		
Item 18	4	3	4	3	4	4	6	1		
Item 19	4	3	4	3	4	4	6	1		
Item 20	4	3	4	3	4	4	6	1		
Item 21	4	3	4	3	4	4	6	1		
Item 22	4	2	4	2	4	4	4	0.67		
Item 23	4	3	4	3	4	4	6	1		
Item 24	4	3	4	3	4	4	6	1		
Item 25	4	3	4	3	4	4	6	1		
Item 26	4	4	4	4	4	4	6	1		
Item 27	4	3	4	3	4	4	6	1		
Item 28	4	4	4	4	4	4	6	1		
Item 29	3	3	4	3	4	2	5	0.83		
Item 30	4	3	4	3	4	4	6	1		
Item 31	4	3	4	3	4	4	6	1		
Item 32	4	3	4	3	4	4	6	1		
Item 33	4	3	4	2	4	4	5	0.83		
Item 34	4	3	4	2	4	4	5	0.83		
Item 35	3	3	4	3	4	3	6	1		
